# Characterization of the complete chloroplast genome of *Hovenia acerba* (Rhamnaceae)

**DOI:** 10.1080/23802359.2020.1714492

**Published:** 2020-01-31

**Authors:** Lin Zhang, Runli Mao, Huitao Bi, Jiemei Shen, Yihan Wang, Mingwan Li

**Affiliations:** a College of Forestry, Henan Agricultural University, Zhengzhou, China;; b Economic Forest and Forest Tree Seedlings Station, Zhengzhou, China;; c College of Life Science, Henan Agricultural University, Zhengzhou, China

**Keywords:** *Hovenia acerba*, chloroplast genome, phylogenetic analysis

## Abstract

*Hovenia acerba* is a widely distributed species with economic, ornamental, and medicinal value in China. In this study, we assembled and characterized the complete chloroplast genome of *H. acerba* for the first time. The circular genome has a quadripartite structure with 161,651 bp in length and contains a pair of 26,619 bp inverted repeat (IR) regions, separated by the large single-copy (LSC, 89,443 bp) region and small single-copy (SSC, 18,970 bp) region. The plastid genome harbours 104 unique genes, including 72 protein-coding genes, 28 tRNAs, and four rRNAs. The overall GC content of the whole genome was 36.7%. Further, the phylogenetic analysis showed that *H. acerba* clustered together with *Ziziphus* genus. The complete chloroplast genome of *H. acerba* will provide important information for phylogenetic and evolutionary studies in Rhamnaceae, as well as the other closely related family.

*Hovenia* Thunb. is a genus of deciduous perennial trees in Rhamnaceae family, including three species and two varieties naturally distributed in China, Bhutan, India, Japan, Korea, Myanmar, and Nepal (Chen and Schirarend 2000). *Hovenia acerba* Lindl., as an important economic, ornamental, and medicinal tree species, has become the most popular and highly valued fruit tree in China. Its hard timber with fine-grained wood is good for making furniture. The dilating peduncles of the infructescence are sweet and edible. In addition, the seeds fruit stems and bark have great medicinal value (Xu et al. 2012; Yang et al. 2013). In this study, we reported the complete chloroplast genome sequence of *H. acerba* for future phylogenetic and genetic studies (GenBank accession number: MN794429).

Fresh leaves of *H. acerba* were collected from Zhengzhou (Henan, China, E113.6253°, N34.7529°, 108 m a.s.l.) and voucher specimens (LMW20190828) were deposited at the Herbarium of Henan Agricultural University. Total genomic DNA was extracted using modified CTAB protocol (Yang et al. 2014). The 150 bp pair-end (PE) reads were generated on the Illumina HiSeq 2500 instruments. The PE reads were assembled using GetOrganelle pipeline (Jin et al. 2018) with *Ziziphus jujube* as the reference. The filtered plastid reads were transferred to the Bandage software (Wick et al. 2015) for viewing and editing. The genome annotation was automatically performed with CpGAVAS (Liu et al. 2012), then adjusted and confirmed the annotated genes using Geneious Prime 2019.2 (https://www.geneious.com/). The physical map of the plastome was drawn with OGDRAW (Lohse et al. 2013).

The complete chloroplast genome of *H. acerba* was a quadripartite circular with 161,651 bp in length, comprising a large single-copy (LSC) of 89,443 bp and a small single-copy (SSC) of 18,970 bp, which were separated by two inverted repeat (IR) regions of 26,619 bp. The base compositions of *H. acerba* chloroplast genome included 31.2% A, 18.0% G, 18.7% C, 32.1% T, with an overall GC content of 36.7%. A total of 104 unique genes were encoded, including 72 protein-coding genes (PCGs), 28 tRNA genes, and four rRNA genes, whereas 18 genes duplicated in the IR regions. Among annotated genes, 12 genes (*trnA-UGC*, *trnI-GAU*, *ndhB*, *rpl2*, *trnK-UUU*, *rps16*, *rpoC1*, *trnL-UAA*, *petB*, *rpl16*, *ndhA*, and *ycf1*) contained a single intron, and three genes (*rps12*, *ycf3*, and *clpP*) had two introns.

In order to determine the phylogenetic position of *H. acerba* in Rhamnaceae family, a maximum-likelihood tree was constructed based on complete chloroplast genome sequences of 10 other Rhamnaceae taxa and five species within a closely related family using RAxML-8.2.12 software (Stamatakis 2014) after the sequences were aligned with MAFFT v7.307 (Katoh and Standley 2013). The results showed that *H. acerba* clustered together with *Ziziphus* genus and was a little far from other genera *Spyridium*, *Berchemiella*, and *Berchemia* in Rhamnaceae family (Figure 1). The complete chloroplast genome of *H. acerba* will provide important information for phylogenetic and evolutionary studies in Rhamnaceae, as well as the closely related family.

**Figure 1.**
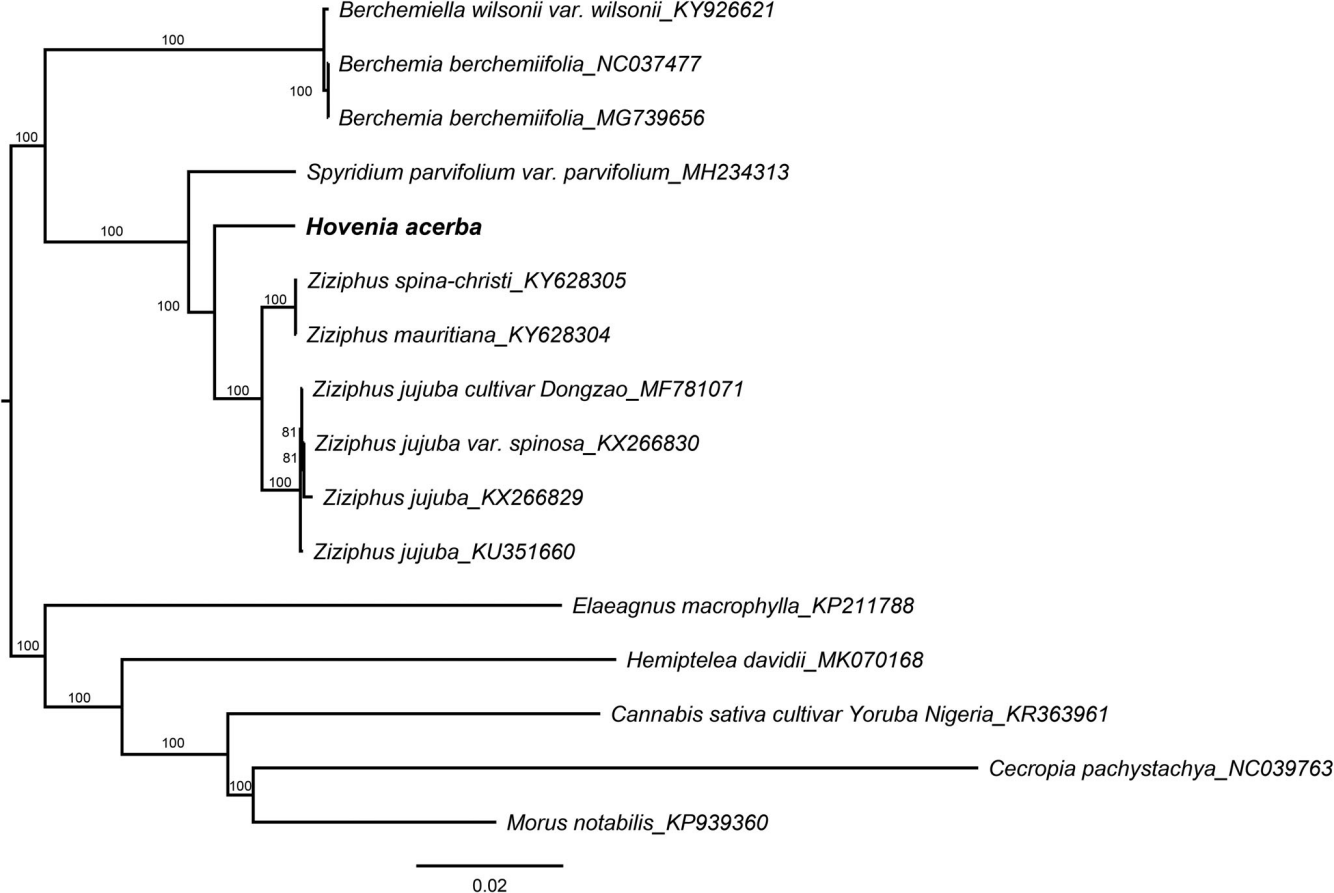
Phylogenetic tree reconstruction of 16 taxa with maximum-likelihood method based on complete chloroplast genomes sequences. Bootstrap values based on 1000 replicates were provided near branches. GenBank accessions are provided after underlines. *Hovenia acerba* was highlighted in bold.
